# Instrumented Mouthguard Decoupling Affects Measured Head Kinematic Accuracy

**DOI:** 10.1007/s10439-024-03550-9

**Published:** 2024-07-02

**Authors:** Ryan A. Gellner, Mark T. Begonia, Matthew Wood, Lewis Rockwell, Taylor Geiman, Caitlyn Jung, Steve Rowson

**Affiliations:** Virginia Tech (Biomedical Engineering and Mechanics), Blacksburg, VA USA

**Keywords:** Instrumented mouthguard, Boil-and-bite, Decoupling, Measurement error, Head, Impact, Kinematics

## Abstract

Many recent studies have used boil-and-bite style instrumented mouthguards to measure head kinematics during impact in sports. Instrumented mouthguards promise greater accuracy than their predecessors because of their superior ability to couple directly to the skull. These mouthguards have been validated in the lab and on the field, but little is known about the effects of decoupling during impact. Decoupling can occur for various reasons, such as poor initial fit, wear-and-tear, or excessive impact forces. To understand how decoupling influences measured kinematic error, we fit a boil-and-bite instrumented mouthguard to a 3D-printed dentition mounted to a National Operating Committee on Standards for Athletic Equipment (NOCSAE) headform. We also instrumented the headform with linear accelerometers and angular rate sensors at its center of gravity (CG). We performed a series of pendulum impact tests, varying impactor face and impact direction. We measured linear acceleration and angular velocity, and we calculated angular acceleration from the mouthguard and the headform CG. We created decoupling conditions by varying the gap between the lower jaw and the bottom face of the mouthguard. We tested three gap conditions: 0 mm (control), 1.6 mm, and 4.8 mm. Mouthguard measurements were transformed to the CG and compared to the reference measurements. We found that gap condition, impact duration, and impact direction significantly influenced mouthguard measurement error. Error was higher for larger gaps and in frontal (front and front boss) conditions. Higher errors were also found in padded conditions, but the mouthguards did not collect all rigid impacts due to inherent limitations. We present characteristic decoupling time history curves for each kinematic measurement. Exemplary frequency spectra indicating characteristic decoupling frequencies are also described. Researchers using boil-and-bite instrumented mouthguards should be aware of their limitations when interpreting results and should seek to address decoupling through advanced post-processing techniques when possible.

## Introduction

Head impact exposure in living volunteers has been reported in the literature for years [1–7]. Head impact sensors characterize both impact frequency and magnitude and have been used primarily in athlete and military populations [8]. Mouthguard- and retainer-style sensors have become increasingly popular because of their potential to couple directly to the upper dentition and, hence, the skull. Some sensors perform well in the lab under ideal conditions, where a tight fit with the lower dentition clamping the mouthguard in place is often used [9–12]. The use of these sensors by volunteers in the field likely differs from the methods used to validate them in a lab for reasons such as sub-optimal fit and the potential for users to have unclenched jaws during a measured head impact [11, 13, 14].

Mouthpieces with good retention to the upper dentition may remain locked to the teeth during a measured acceleration event. Still, the forces associated with an impact can be large enough to overcome this coupling force, leading to decoupling of the mouthguard from the teeth. In addition, mouthpiece wear and tear may lower protective effects [15] and teeth retention force over time. Because these devices are often used in fast-paced environments, users may communicate with teammates or fail to anticipate impacts, inevitably resulting in some impacts occurring while users’ mouths are open. Impacts occurring concurrently with users’ mouths being open can lead to data artifacts [11, 16] or complete decoupling. The effects of mouthguard decoupling are challenging to predict in advance and detrimental to research studies using the devices due to data loss, poor data quality, and unexplainable data artifacts. Some mouthpiece sensors include algorithm- or hardware-based switches that disable data recording completely if the mouthpiece is not determined to be in a mouth [13, 17]. These switches may cause the mouthguard to forego measurement if enough relative movement occurs before or during an impact, possibly missing critical head impacts associated with injury. If data are captured, noise artifacts may cause erroneously high measurements in linear or angular kinematics as the mouthguard’s small mass and moment of inertia relative to the head encourage higher accelerations when not rigidly attached to the teeth [18, 19].

If researchers can identify mouthguard decoupling in field impacts, the next hurdle is finding the best method of dealing with them. Strict removal of affected impacts is the most straightforward answer, but this reduces sample size, introduces sampling biases, and decreases the chance of catching the most critical impacts—those rare events associated with head injury [18]. Thus, the ability to salvage affected impacts would be the most valuable method of dealing with decoupling events. While this may not be possible for all decoupling events, minimizing error in as many impacts as possible is crucial to understanding head impact biomechanics and injury tolerances in human volunteer studies.

This study aimed to determine the effects of instrumented mouthpiece decoupling on measurement error, as compared to a ground truth measurement placed at a headform center of gravity (CG). We also sought to quantify signal characteristics associated with decoupling. Here, we define decoupling as the mouthpiece sensor moving relative to the teeth. The hypothesis was that the mouthguard would become less accurate with an increased gap from the lower jaw to the mouthguard, which corresponded to increasing movement relative to the headform’s dentition.

## Methods

Laboratory head impact tests were conducted using a pendulum impactor and a padded impactor face. A medium National Operating Committee on Standards for Athletic Equipment (NOCSAE) headform housed laboratory-grade instrumentation at its CG and a commercially available instrumented mouthguard on its teeth. We obtained raw mouthguard signals, which we then filtered and transformed to the headform CG for comparison. We calculated error by comparing the peak values of the CG-transformed linear accelerations to those measured directly at the CG. Measurements collected at the CG were also inversely transformed to the teeth location to generate relative acceleration signals to identify decoupling. Decoupling signals were calculated as the difference in kinematic signal traces. We rotated all measurements to match the coordinate system designated by the Society of Automotive Engineers (SAE) with the x-axis in the posterior-anterior direction, y-axis in the medial-lateral direction, and z-axis in the superior-inferior direction [20].

**Fig. 1:**
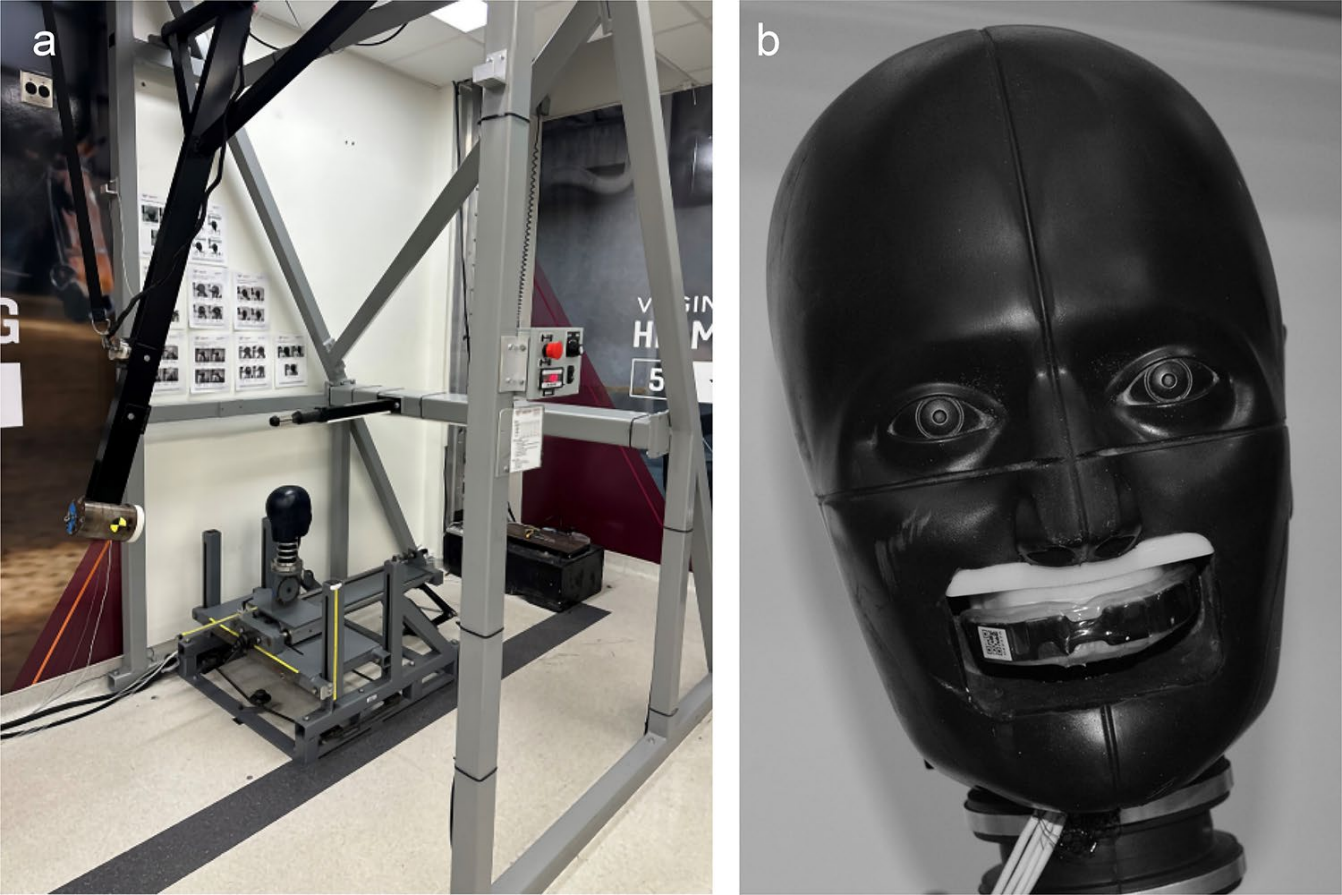
**a** Gravity-driven pendulum impactor used to accelerate headform. **b** NOCSAE headform with 3D-printed dentition and boil and bite mouthguard in place (lower dentition plate not shown)

We used a pendulum to impact the bare medium NOCSAE headform mounted on a 50th percentile male Hybrid III neck [21, 22], which was free to slide along a single axis of a 5-degree-of-freedom linear slide table (Fig. 1A). Via a headform modification, 3D-printed dental arches were mounted in an anatomically correct location for mouthguard testing (Fig. 1B) [1]. We instrumented the headform with three linear accelerometers (Endevco 7264b-2000) and a triaxial angular rate sensor (DTS ARS3 Pro 18k) at the headform’s CG. Headform data were collected at 20 kHz for 150 ms, including 50 ms pre-trigger and 100 ms post-trigger. Data collection was triggered when the dominant axis exceeded 5 g (e.g., the *x*-axis for Front location impacts)*.* The sampling rate of the lab instrumentation was 20 kHz, and the data collection system applied a hardware anti-aliasing filter with a cutoff frequency of 4 kHz to all channels before any other operations.

**Fig. 2:**
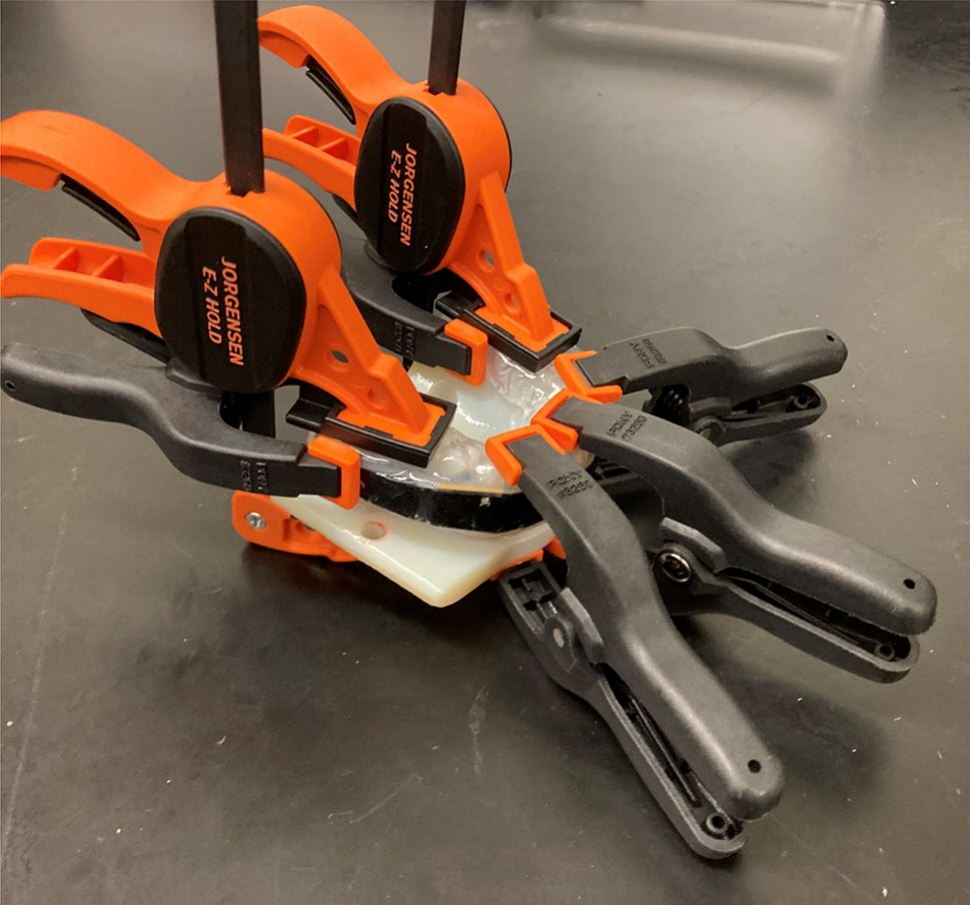
Spring clamps provided “bite” force for fitting the boil-and-bite mouthguard to the 3D-printed dentition

We modified the NOCSAE headform so that 3D-printed dental arches could be mounted in an anatomical location for this test series [9]. Relative to the headform CG, the mouthguard instrumentation was at + 82 mm along the *x*-axis, − 9 mm along the *y*-axis, and + 65 mm along the *z*-axis. We fitted a boil-and-bite style mouthguard (Prevent Biometrics, Edina, MN) to the dentition. The manufacturer’s instructions required boiling the mouthguard for 20 s, followed by placing the mouthguard on the teeth. The user is then told to bite under suction for 30 s, followed by biting for an additional 30 s. We had to adapt the instructions for our dentition, as suction could not be applied. Instead, we used multiple clamps to provide force around the perimeter of the mouthguard. Five spring clamps and two trigger clamps provided clamping force (Pony Jorgensen, Saddle Brook, NJ—Fig. 2). We used the manufacturer's recommended times for boiling and fitting. We confirmed that fit was acceptable per the manufacturer’s standards by ensuring the mouthguard passed the “open mouth” test—it remained on the teeth under an open-mouth condition by pressing it onto the teeth and then letting go. The mouthguard did not fall off the teeth when no external pressure was applied. During testing, we placed an aluminum plate at varying distances from the bottom surface of the mouthguard to create a “closed mouth” condition and two “open mouth” conditions.

The mouthguard was instrumented with a three-axis accelerometer and a three-axis gyroscope, each sampling at 3.2 kHz. For the given sampling rate, the linear accelerometer had a bandwidth of 1600 Hz, while the gyroscope had a bandwidth of 890 Hz—both above the frequencies of interest [23]. Mouthguard data collection was triggered when any one accelerometer channel exceeded 8 g. The measurement window included 50 ms of data, including 10 ms of pre-trigger data and 40 ms of post-trigger data. The mouthguard output was tailored such that all raw data were exported, enabling the authors to do the signal processing entirely. No data post-processing techniques typically implemented within the mouthguard manufacturer’s data download system were employed for this study.

**Fig. 3:**
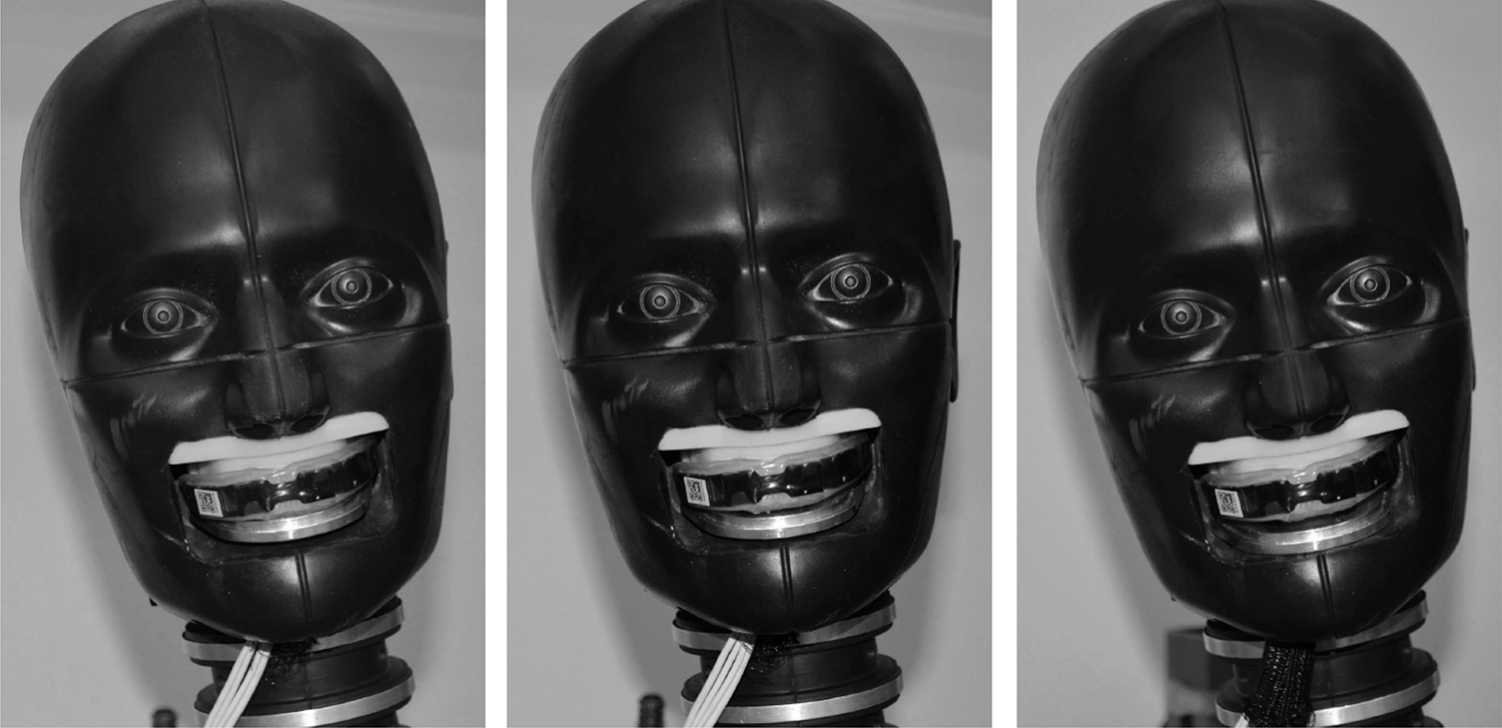
Lower dentition to mouthguard gap conditions from left to right: 0 mm, 1.6 mm, and 4.8 mm

The pendulum impactor was covered with a vinyl nitrile foam pad (VN600, Dertex Corporation) or a rigid nylon face. The padded impactor face generated a resultant linear acceleration duration of approximately 10–12 ms, while the rigid face generated durations of approximately 3–5 ms. Four locations were impacted on the headform (front, front boss, rear, and rear boss) at four different pendulum angles, as described in detail in previous studies [9]. These pendulum angles corresponded to four target peak resultant linear head acceleration severities (25, 50, 75, and 100 g). Peak linear acceleration targets were said to have been achieved if the impact resulted in a peak resultant linear acceleration at the headform CG of plus or minus 10% of the target linear acceleration. We tested three decoupling conditions for each combination of location and severity: no gap, slight gap, and large gap. The gap conditions were introduced by tightening the bottom aluminum dentition to the lower face of zero, one, or three 1.6 mm shims stacked on top of one another, resulting in a no-gap condition of 0 mm or a gap condition of either 1.6 mm or 4.8 mm (Fig. 3). The shims were removed after setting the gaps. This setup created a preset, fixed opening between the mouthguard and the lower dentition in the gap conditions. The mouthguard stayed attached to the upper dentition until the time of impact in all tests, as verified by high-speed video. Each configuration underwent two trials for a total of 192 tests.

Some rigid impacts were not adequately collected or were missed entirely by the mouthguard sensors. This data loss could have been due to inherent limitations with the mouthguard instrumentation (e.g., sampling rate) or due to data rejection algorithms within the mouthguard firmware (version 2.0.14). Sometimes, the mouthguard missed the primary impact but collected a secondary impact (e.g., when the headform carrier slid into the spring stopper at the end of the track). To ensure these data artifacts did not influence the results, we excluded impacts wherein the mouthguard missed the primary impact (32 impacts, 17%; Table 1).

**Table 1:** Number of trials collected in each impact configuration

Impactor face	Impact direction	Gap condition (mm)	25 g target linear accel	50 g target linear accel	75 g target linear accel	100 g target linear accel
Pad	Front	0	2	2	2	2
		1.6	2	2	2	2
		4.8	2	2	2	2
	Front boss	0	2	2	2	2
		1.6	2	2	2	2
		4.8	2	2	2	2
	Rear	0	2	2	2	2
		1.6	2	2	2	2
		4.8	2	2	2	2
	Rear boss	0	2	2	2	2
		1.6	2	2	2	2
		4.8	2	2	2	2
Rigid	Front	0	2	2	**1**	2
		1.6	2	**1**	2	**1**
		4.8	2	**1**	***0***	***0***
	Front boss	0	2	***0***	***0***	***0***
		1.6	2	***0***	***0***	***0***
		4.8	2	***0***	***0***	***0***
	Rear	0	2	2	2	2
		1.6	2	2	2	2
		4.8	2	2	2	2
	Rear boss	0	**1**	2	2	2
		1.6	**1**	2	2	***0***
		4.8	**1**	2	2	**1**

Bold cells denote one of two trials were missed. Bold italics cells denote two of two trials were missed

Raw data output by the mouthguard sensors were filtered using a second-order phaseless Butterworth dual-pass filter [24, 25] and transformed to the headform CG using rigid body dynamics equations (Eq. 1). Angular acceleration was calculated via a five-point stencil from filtered angular velocity measurements [26]. Based on previous analyses, filter cutoff frequencies (− 3 dB point) were chosen for the mouthguard data: in padded impacts, 100 Hz for linear accelerometers and 175 Hz for angular rate sensors; in rigid impacts, 175 Hz for linear accelerometers and 275 Hz for angular rate sensors. These filters best match ground truth CG measurements filtered to SAE J211-1 recommended values [27]. Data collected at the headform CG were filtered using SAE J211 recommended filter cutoff frequencies: 1650 Hz, equivalent to CFC1000, was used for linear accelerometers and 300 Hz, equivalent to CFC180, was used for angular rate sensors [20, 28].

1 $$\overrightarrow {{a_{{{\text{CG}}}} }} = \overrightarrow {{a_{P} }} + \vec{\omega } \times \left( {\vec{\omega } \times \vec{r}} \right) + \vec{\alpha } \times \vec{r}$$

where *a*_*CG*_ is linear acceleration at the CG, *a*_*P*_ is linear acceleration at the mouthguard, *r* is the vector distance from point CG to point *P*, $\omega$ is angular velocity, and $\alpha$ is angular acceleration of the rigid body.

Error was computed as the difference between the peak values of the mouthguard signals and the headform CG measurements for both kinematic measurements (linear acceleration and angular velocity) and the calculated value of angular acceleration. We computed percent error by dividing magnitude error by the CG measurement (Eq. 2). We analyzed error with mouthguard peak values reported from the entire time window available from the mouthguard traces (50 ms), as this peak value is what would be reported in the field for a given impact. We noticed that relative differences between the reference and mouthguard signals occurred later in the data collection window; therefore, we repeated our analyses using mouthguard-reported peak values found only within the impact duration. Impact duration was defined as 12 ms for padded impacts and 5.5 ms for rigid impacts, to account for variation in linear acceleration duration by location. We searched the impact from − 1 to 5.5 ms for rigid impacts and − 1 ms to 12 ms for padded impacts, relative to the trigger at time 0 ms, to account for slight variations in trigger and peak timing within impacts.

2 $$\in_{\% } = \frac{{{\text{PK}}_{{{\text{MG}}}} - {\text{PK}}_{{{\text{CG}}}} }}{{{\text{PK}}_{{{\text{CG}}}} }} \times 100\%$$

where $\in_{\% }$ is percent error, PK_MG_ is the peak of the kinematic signal from the mouthguard measurement, and PK_CG_ is the peak of the kinematic signal from the measurement at the CG.

To test our hypothesis that the mouthguard would become less accurate with an increasing gap, we used a linear model, performed an ANOVA on the model, and also tested contrasts between factor variables using least squares means. We included gap condition, target linear acceleration (impact severity), impactor face (impact duration), and general location (frontal: front and front boss locations, or rearward: rear and rear boss locations) as independent variables in our linear model. The response variables were the percent errors in peak kinematics from each of the three kinematic variables. We repeated this using the duration-windowed dataset, for a total of six linear models.

Additionally, we used weighted least product regression (WLP) analysis with bootstrapped 95% confidence intervals to determine whether fixed bias, proportional bias, or both were present with each mouthguard condition relative to CG measurements [29, 30]. Least product regression considers error in both *x*- and *y*-values when calculating the line of best fit. We used least product regression because although the laboratory measurement was considered the gold standard, some variance exists in the laboratory measurements. We used weighted regression (rather than ordinary) to account for the observed heteroscedasticity. We bootstrapped the data to 10,000 replicates to generate 95% confidence intervals. Fixed bias occurs when the 95% confidence interval for the WLP regression line’s intercept does not include zero: this means the measurements are consistently higher or lower than the reference value. Proportional bias measures for the inclusion of one in the 95% confidence interval for the WLP line’s slope. If one is not included, the measurements are proportionally higher or lower than the reference value. Both can be present in the same dataset.

Headform data collected at the CG were also inversely transformed to the headform dentition location using rigid body dynamics. This enabled us to calculate relative acceleration of the mouthguard with respect to the teeth. We filtered raw laboratory data using CFC180 for both linear acceleration and angular velocity for maximum accuracy in transformation-based 6DOF measurements [25]. We downsampled laboratory data (MATLAB: *resample*) to match the mouthguard sampling rate, and the mouthguard and laboratory signals were time-aligned at the point of maximum correlation using cross-correlation (MATLAB: *xcorr*). We visually confirmed alignment for all signal pairs. Laboratory and mouthguard data were filtered to CFC 180 (− 3 dB point of 300 Hz). We chose this cutoff frequency because it was the optimal for transforming lab measurements to the teeth and we wanted frequency attenuation to be equal across measurement devices [25]. We calculated relative linear acceleration and relative angular velocity as the differences between mouthguard and headform measurements at the teeth location. We calculated relative angular acceleration as the difference between the derived angular acceleration signals from each sensor package. Individual axis measurement and the resultant signals were time-aligned and subtracted (mouthguard minus CG) to generate relative motion along each axis.

We transformed relative motion time history curves to the frequency domain using a Fast Fourier Transform (FFT, MATLAB: *fft*). Relative motion curves were zero-padded to five times their original length to increase visual frequency resolution. An FFT was run on the zero-padded curves to generate frequency domain information for each curve. A single-sided FFT, up to the Nyquist frequency (1600 Hz), was used. We calculated amplitude by dividing the FFT output by the length of the input signal, taking the absolute value, and multiplying by two.

We completed all data processing in MATLAB R2023a and R2023b (Mathworks—Natwick, MA). We completed the statistical analysis (including bootstrapping) and visualization using RStudio 2023.06.0 (R Foundation for Statistical Computing, Vienna, Austria). We used a predetermined level of *p* = 0.05 for significance in our linear models.

## Results

**Fig. 4:**
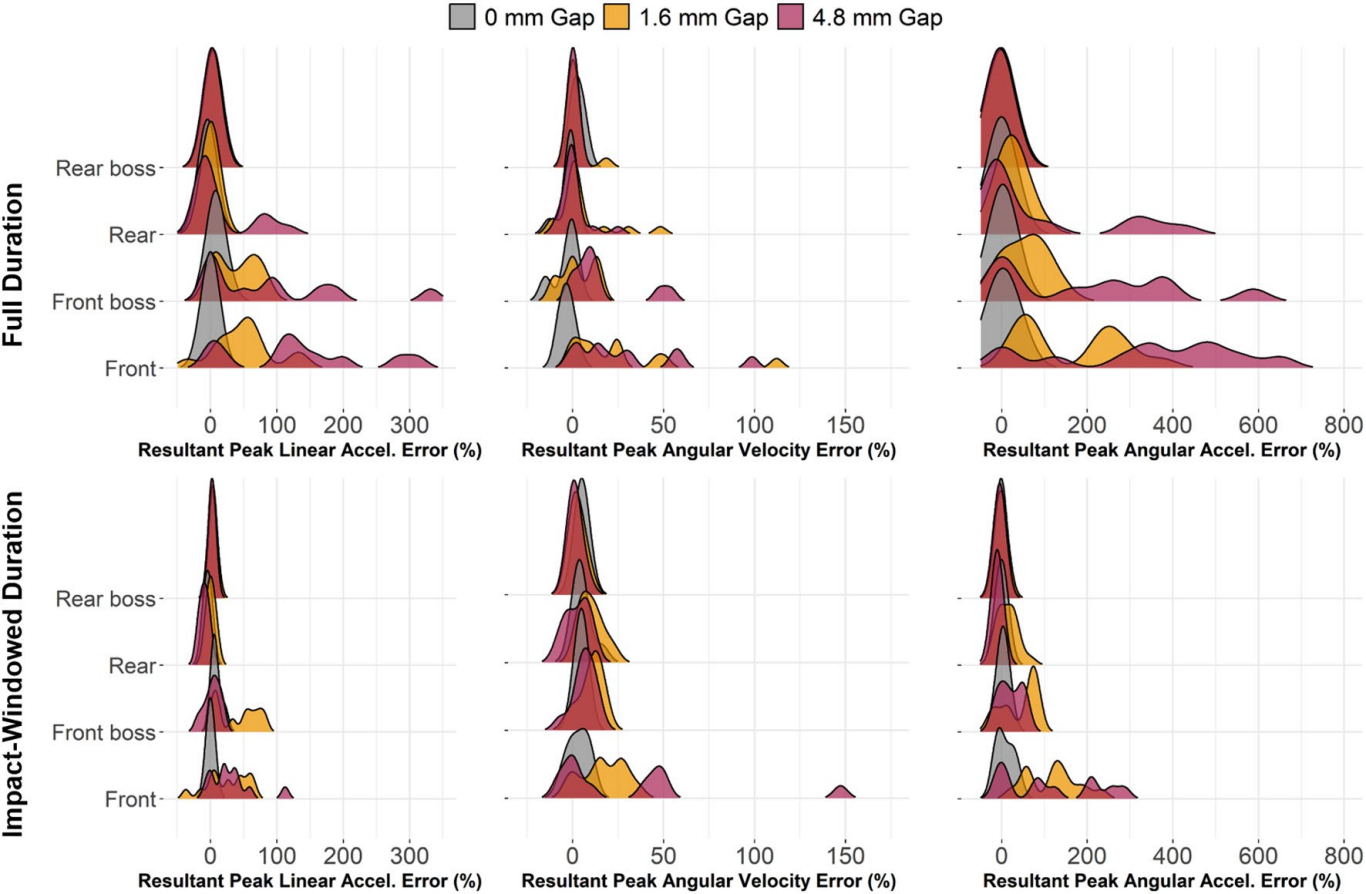
Probability distributions of percent error for each kinematic by impact location and duration. Differences in error by impact location were observed regardless of gap size, with differences becoming more pronounced with increases in gap size. Front and front boss impacts tended to exhibit wider error distributions than rear and rear boss impacts. Windowing the signals to the impact duration resulted in tighter error distributions

The major factors influencing mouthguard measurement error were gap size, impact direction, and impactor face. Larger gaps in frontal impacts (front and front boss) generally produced greater errors (Fig. 4). Rigid impacts had less error than padded impacts, but this was likely influenced by the missing rigid impacts, which were generally frontal impacts of higher severity. Fixed and proportional bias were found in mouthguard measurements; these generally decreased in severity or were eliminated with less decoupling (smaller gap) and when the analysis was limited to the impact duration only. Lastly, decoupling signals were characterized by their time- and frequency-signals. Specific axes were shown to have higher frequency amplitudes in relative motion signals when decoupling was present.

### Average Error in Full Measurement Duration Analysis

We first quantified measurement error and bias for peak kinematics by using the entire duration of measurement reported by the mouthguard (10 ms pre-trigger to 40 ms post-trigger) because in typical use, instrumented mouthguards report peak kinematics over the entire collection window. We combined the front and front boss locations (Frontal) and rear and rear boss locations (Rearward) because these impact types had similar error trends. In general, mean error and variance increased with gap size for each kinematic measure (Table 2).

**Table 2:** Differences in percent error in peak resultant kinematics for full measurement window

Impactor face	Impact direction	Gap condition (mm)	Peak linear accel	Peak angular velocity	Peak angular accel
			Percent error (%)* ^†‡^	Percent error (%)*^†‡^	Percent error (%)*^†‡◊^
Pad	Frontal	0	1.9 ± 4.8	− 5.0 ± 4.7	− 8.1 ± 10.2
		1.6	54.8 ± 29.9	11.2 ± 17.7	116.0 ± 95.9
		4.8	140.0 ± 105.0	21.3 ± 21.8	332.0 ± 213.0
	Rearward	0	0.4 ± 4.0	− 1.7 ± 4.4	− 5.3 ± 3.5
		1.6	− 0.1 ± 4.7	− 0.8 ± 4.2	10.6 ± 30.7
		4.8	23.3 ± 41.4	− 0.8 ± 2.9	82.5 ± 164.0
Rigid	Frontal	0	6.2 ± 9.5	− 0.4 ± 3.2	24.6 ± 8.8
		1.6	28.6 ± 52.1	43.5 ± 64.9	124.0 ± 141.0
		4.8	26.6 ± 48.9	28.1 ± 39.7	91.5 ± 119.4
	Rearward	0	− 2.5 ± 7.1	1.7 ± 3.7	4.2 ± 4.3
		1.6	4.9 ± 4.4	9.1 ± 15.5	17.4 ± 13.3
		4.8	− 4.9 ± 9.8	2.1 ± 7.5	7.05 ± 38.6

ANOVA results: *significantly different by gap size

^†^Significantly different by impact direction

^‡^Significantly different by impactor face

^◊^Significantly different by impact severity

Percent error in peak linear acceleration was significantly influenced by the gap size (*p* < 0.001), impactor face (*p* = 0.001), and impact direction (*p* < 0.001). Each 1 mm increase in gap size was associated with 11% greater error. Padded impacts had an average of 25% greater error than rigid. Rearward impacts generated 46% less error on average.

The model for peak angular velocity percent error showed significant influence from gap size (*p* = 0.008), impactor face (*p* = 0.019), and impact direction (*p* < 0.001). A gap size increase of 1 mm corresponded to a 2% increase in error for peak angular velocity. Padded impacts generated 7.9% lower error than rigid impacts, on average. Impacts to the front portion of the headform (front and front boss) had 13% higher error than rearward directed (rear and rear boss) impacts.

Finally, the linear model with angular acceleration percent error as its response variable showed significance for gap size (*p* < 0.001), impactor face (*p* = 0.001), impact direction (*p* < 0.001), and impact severity (*p* = 0.005). An increase in gap size by 1 mm generated an average of 29% greater error in angular acceleration. Padded impacts generated 37% greater error than rigid impacts, on average. Impact direction had the greatest effect on error, with impacts to the front or front boss locations generating 107% greater error than those to the rearward locations on average. Every 1 g increase in impact severity corresponded to a 0.9% increase in peak angular acceleration error.

### Average Error in Impact Duration-Windowed Analysis

Measurement error and bias were also quantified for peak kinematics during the duration of impact only. Impact duration was defined as − 1 to 5.5 ms for rigid impacts and − 1 ms to 12 ms for padded impacts, relative to trigger. We chose these times so that impact-relevant peaks were captured (see example time history plots, Fig. C1). Impact-relevant peaks were defined based on the lab instrumentation measurements of linear acceleration for each impact because the lab instrumentation was not subject to decoupling conditions. Linear acceleration was chosen because it was the best indicator of the duration of force input to the head.

After windowing the signals to include only the impact duration, mean error and variance did not always increase with gap size (Table 3). In general, the magnitudes of errors were much lower after windowing in the full measurement duration analysis. Resultant peak linear acceleration percent error was significantly associated with impactor face (*p* = 0.003) and the impact direction (*p* < 0.001), but not gap size (*p* = 0.619) or impact severity (*p* = 0.91). Padded impacts had, on average, 9% greater error in peak resultant linear acceleration. Frontal impacts generated 17% higher error per impact than rearward.

**Table 3:** Differences in percent error in peak resultant kinematics for impact duration window

Impactor face	Impact direction	Gap condition (mm)	Peak linear accel Percent error (%)^†‡^	Peak angular velocity Percent error (%)^†^	Peak angular accel Percent error (%) *^†^◊
Pad	Frontal	0	1.9 ± 4.8	2.6 ± 4.0	− 3.9 ± 6.2
		1.6	44.8 ± 25.1	11.4 ± 9.7	87.6 ± 58.8
		4.8	14.7 ± 20.7	10.8 ± 18.5	63.6 ± 87.2
	Rearward	0	0.4 ± 3.7	6.4 ± 4.4	− 5.3 ± 3.5
		1.6	− 0.1 ± 4.7	5.9 ± 4.4	− 1.2 ± 19.4
		4.8	− 1.3 ± 4.5	5.1 ± 3.7	− 8.1 ± 3.9
Rigid	Frontal	0	6.2 ± 9.5	5.7 ± 5.0	24.6 ± 8.8
		1.6	2.6 ± 22.7	19.7 ± 10.6	86.4 ± 81.3
		4.8	26.6 ± 48.9	43.9 ± 60.0	90.1 ± 120.6
	Rearward	0	− 2.5 ± 7.1	3.7 ± 2.3	4.2 ± 4.3
		1.6	4.9 ± 4.4	8.0 ± 8.3	17.4 ± 13.3
		4.8	− 7.1 ± 9.4	− 1.5 ± 4.1	− 6.8 ± 7.8

ANOVA results: *significantly different by gap size

^†^Significantly different by impact direction

^‡^Significantly different by impactor face

^◊^Significantly different by impact severity

In the impact duration-windowed model, peak angular velocity percent error had even fewer significant factors than linear acceleration: only impact location (*p* < 0.001) was significantly associated with error in angular velocity. Frontal impacts generated 7.6% greater error than rearward impacts for peak angular velocity.

The final linear model revealed significant association between percent error in peak angular acceleration and gap size (*p* = 0.028), impact direction (*p* < 0.001), and impact severity (*p* = 0.001). Every 1 mm increment in gap size corresponded to a 4% increase in peak angular acceleration error. Frontal impacts generated 56% higher errors, on average. And each 1 g increase in impact severity was associated with 0.43% greater error in peak angular acceleration.

### Weighted Least Products Bias

**Fig. 5:**
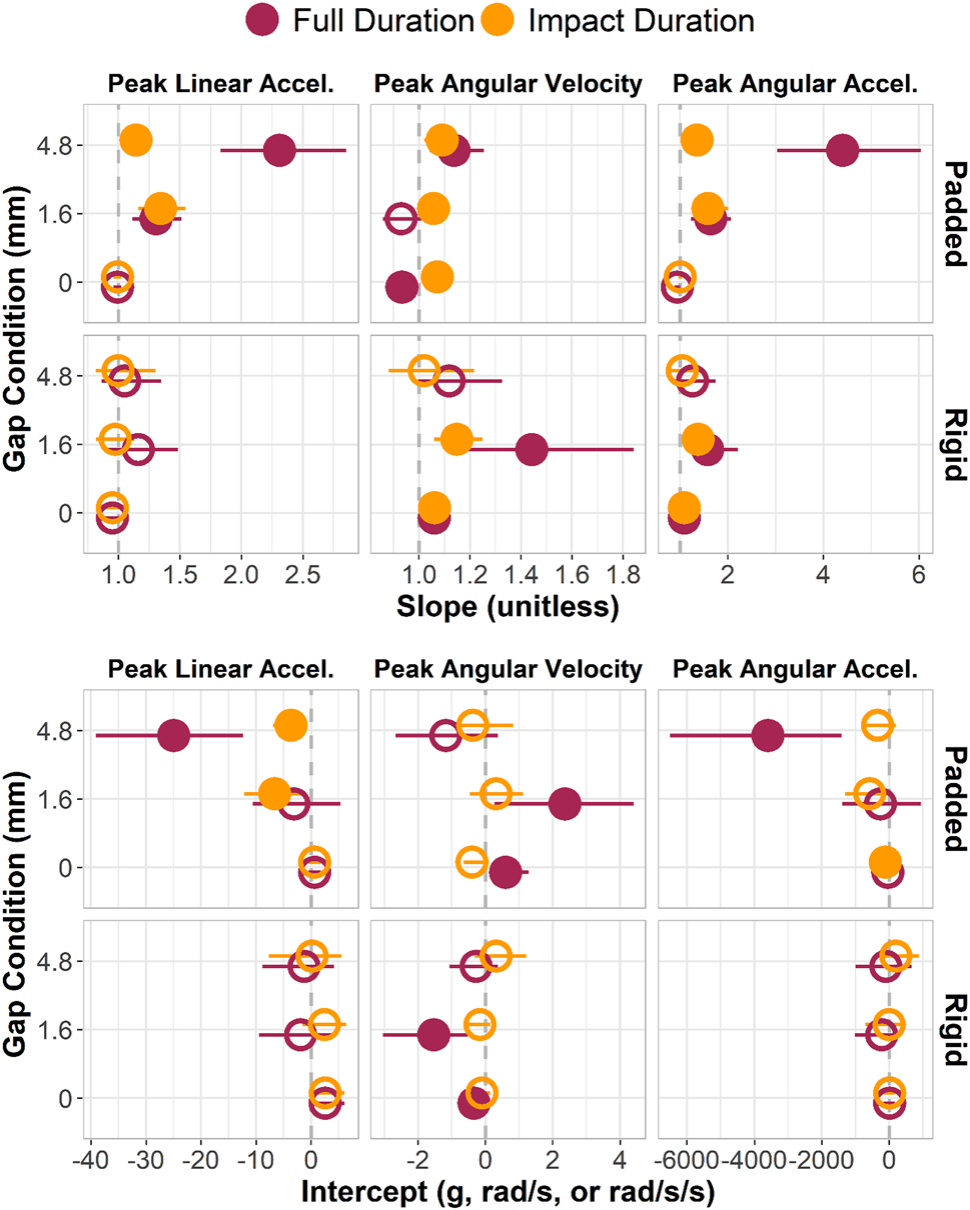
Weighted least product slope and intercept estimates and bootstrapped confidence intervals. Gap conditions generally had slope values further from one and intercept values further from zero. Limiting the analysis window to the impact duration (orange dots) generally produced slopes closer to one and intercepts closer to zero, especially in the 1.6 mm and 4.8 mm gap conditions. Filled points indicate statistically significant proportional (slope) or fixed (intercept) bias

Weighted least product (WLP) regression revealed fixed and proportional bias among mouthguard measurements relative to headform measurements for the same impacts, as determined by the 95% confidence intervals (Fig. 5). Interestingly, both proportional bias (slope) and fixed bias (intercept) severity decreased in most cases when changing from the full impact duration to the impact-windowed analysis, even if the WLP confidence intervals did not show that the bias disappeared with this change. Severity of bias was also generally greater for the 1.6 mm and 4.8 mm gap conditions than for the no gap condition.

Over the full measurement duration window, padded impacts displayed proportional bias (slope ≠ 1) for all gap sizes except for peak linear acceleration (PLA) at 0 mm gap, peak angular velocity (PAV) at 1.6 mm gap, and peak angular acceleration (PAA) at 0 mm gap. Fixed bias existed (intercept ≠ 0) in the largest gap condition in PLA and PAA, but in 0 mm and 1.6 mm gap condition for PAV. Rigid impacts displayed proportional bias only in angular measures: 0 mm and 1.6 mm in PAV, and 0 mm and 1.6 mm in PAA. Fixed bias existed for only 0 mm and 1.6 mm conditions in PAV for rigid impacts.

In the impact duration window, padded impacts displayed proportional bias for all conditions except for PLA 0 mm gap and PAA 0 mm gap. Fixed bias existed in the 1.6 mm and 4.8 mm conditions for PLA, but only in the 0 mm condition for PAA; PAV displayed no fixed bias for padded impacts in the impact duration windowed data. Rigid impacts displayed proportional bias for only angular measures: 0 mm and 1.6 mm in PAV and PAA. Fixed bias existed for no conditions when limiting the analysis to the duration window in rigid impacts. Full scatter plots with WLP lines and confidence intervals can be seen in Appendix 1.

### Decoupling Signatures

**Fig. 6:**
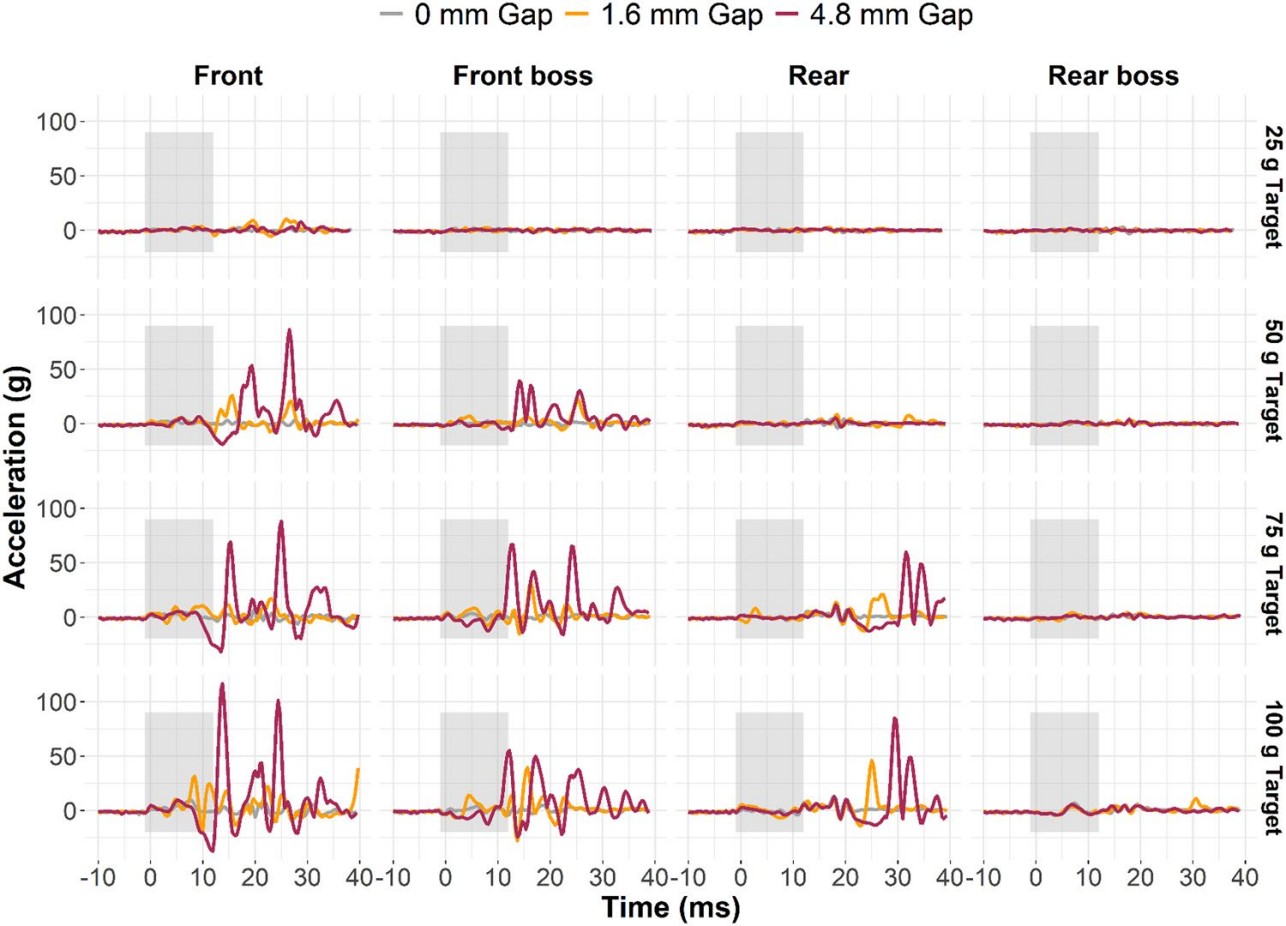
Exemplar average relative linear acceleration time traces at the teeth for each gap condition in padded impacts. Most of the relative motion occurred after the impact event was over (indicated by the gray box). Front and front boss impacts had considerably more relative motion, on average, than rear and rear boss impacts

Next, we analyzed decoupling signal characteristics by subtracting optimally aligned headform signals from mouthguard signals. Differences between measurement signals revealed relative motion artifacts in the mouthguard signals for gap conditions. These relative motion artifacts were increasingly recognizable in the mouthguard measurements as impact severity and lower dentition gap increased (Fig. 6) and were observed in both linear and angular measurements.

**Fig. 7:**
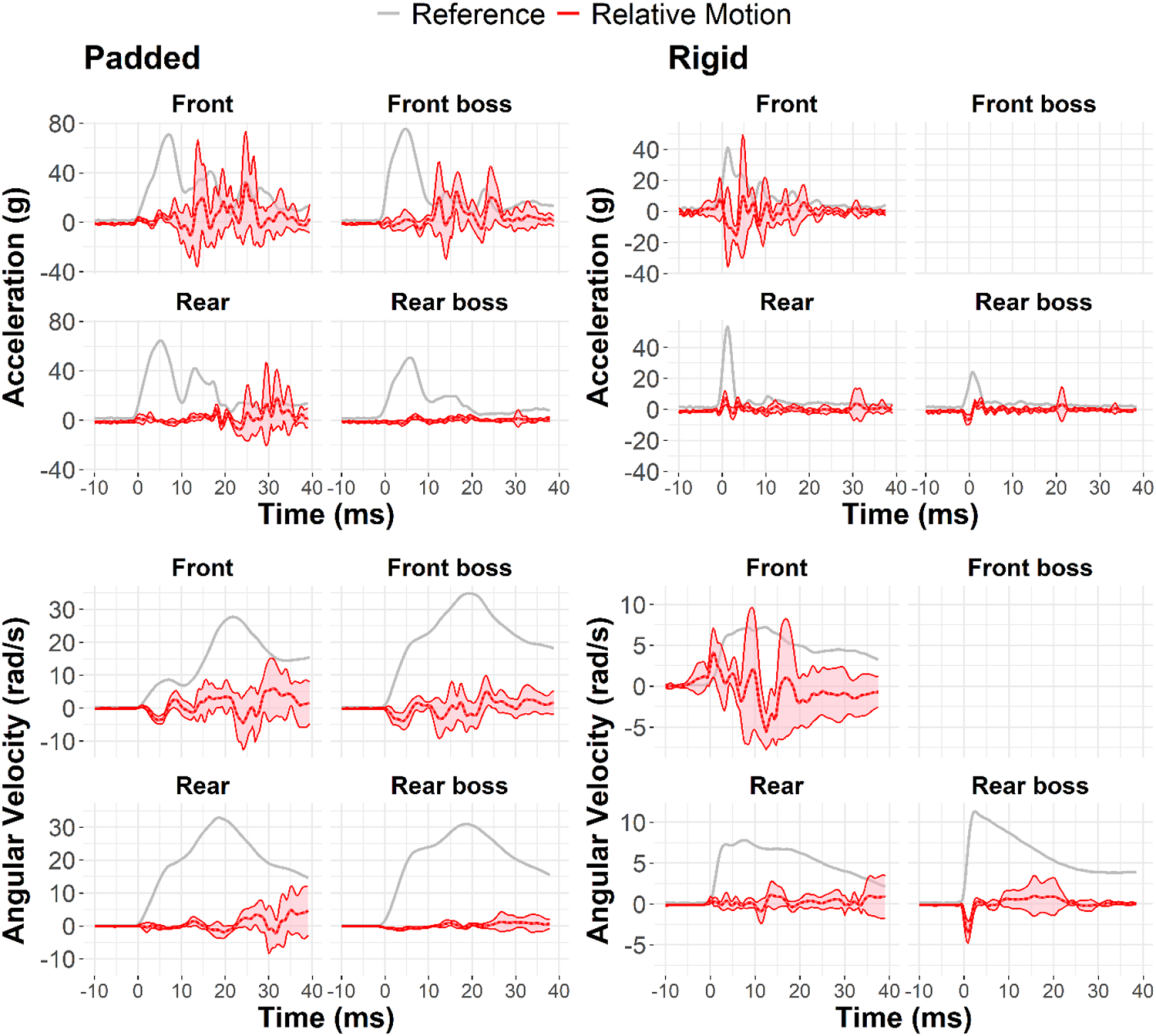
Relative linear acceleration (top row) and angular velocity (bottom row) corridors at the teeth for padded (left column) and rigid (right column) impacts, averaged across both gap conditions and all severities ≥ 50 g resultant linear acceleration (non-trivial motion). Each padded corridor includes data from 12 impacts. The rigid front corridor includes 5 impacts, rigid rear includes 12 impacts, and rigid rear boss includes 9 impacts. The mouthguard did not collect any front boss impacts at ≥ 50 g target severity

Each impact severity and gap condition combination had unique relative motion signal characteristics. Most of the relative linear acceleration was isolated to after the impact had concluded (approximately 10 ms post-trigger in all impacts). Angular velocity continued to increase after this time point because, once up to speed, the head continued to rotate even after the applied force to the head (i.e., the pendulum impactor) was no longer acting on the head. We generated characteristic decoupling signature corridors by using the average and standard deviation of all impacts with non-trivial relative motion (greater than or equal to 50 g target linear acceleration) for the two gap conditions. These corridors were generated for linear and angular measures (Fig. 7).

### Decoupling Spectra

**Fig. 8:**
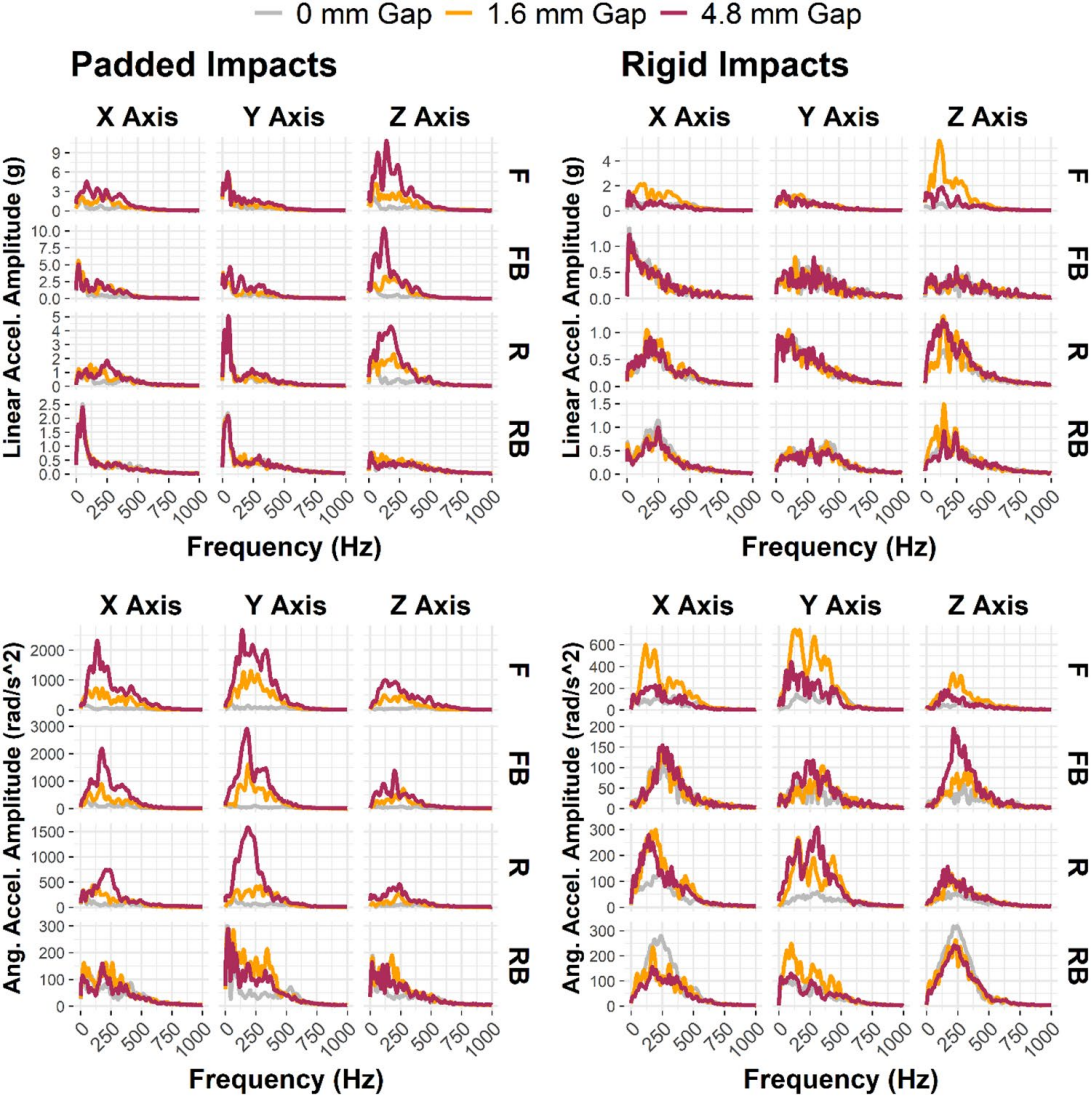
Frequency spectra of padded (left column) and rigid (right column) relative motion impact kinematics. Linear acceleration (top row) showed the highest frequency content along the *Z*-axis in decoupling relative to the no-gap condition. Angular acceleration (bottom row) *X*- and *Y-*axes had the highest frequency content in decoupling, compared to the 0 mm gap condition. *F* front, *FB* front boss, *R* rear, *RB* rear boss

We noted specific frequency bands associated with decoupling that were not present in tight condition impacts from relative motion data. Linear acceleration along the *z*-axis had consistently higher frequency content above 100 Hz for gap condition tests. Additionally, *X* and *Y* channels from angular acceleration showed higher frequency content above 100 Hz when a gap condition was tested (Fig. 8).

## Discussion

This study sought to determine if lower dentition gap, which elicited mouthguard decoupling, affected measurements from instrumented mouthpieces. In general, increasing gap size caused an increase in relative motion between the mouthguard and the headform teeth, which led to a corresponding increase in error for reported peak kinematics from the mouthguard. When decoupling occurred, impacts to the front and front boss locations were characterized by higher relative motion and errors than impacts to the rear and rear boss locations. Specific signal characteristics exist for the decoupling of instrumented mouthpieces, which are generally recognizable by impact location and contaminate the signal with higher frequency content as gap size and relative motion increase.

Recent studies have reported finding similar frequency content caused by decoupling events in instrumented mouthguards used in the field. Luke et al. described ice hockey and rugby field events categorized into one of four categories describing the pre- and post-impact coupling status [13]. Luke combined three categories (decoupled, decoupling, and recoupling) into a poorly coupled group and compared them to the fourth category (coupled). Poorly coupled impacts had significantly higher content in medium (50–200 Hz) and high (> 200 Hz) frequency ranges than coupled impacts for linear and angular acceleration. Our study agrees with this finding: decoupled impacts in a laboratory setting also exhibit higher frequency magnitudes in the frequency ranges described by Luke et al. Additionally, Wu et al. described instrumented mouthguard frequency response in a volunteer soccer heading experiment [14]. These impacts were under confirmed coupled conditions, as the volunteer had clenched teeth. In their study, instrumented mouthguard linear acceleration traces for these coupled impacts produced most of their frequency content below 100 Hz. Our study further corroborates Wu’s results for coupled impacts.

Kuo et al. observed the effects of mandible motion on instrumented mouthguard error [11]. Their study included a clenched jaw, an unconstrained articulating jaw, and a no-jaw condition. They used custom-fit mouthguards, which produced little or no relative motion in the no-jaw condition. The difference in relative motion findings suggests that custom-fit mouthguards (used in Kuo et al.’s study) may have better coupling and less error caused by relative motion than boil-and-bite mouthguards (used in our study). Kuo et al. also found that impact location influenced error, with impacts to the front and top of the head having greater errors than other locations. Our study shows similar location dependence. Finally, Kuo et al. identified characteristic frequency content in error signals from gyroscopes in instrumented mouthguards. Thus, their study presented characteristic frequency content from an unconstrained lower jaw moving into an instrumented mouthguard during impact, while our study presents the characteristic frequency content caused by decoupling and relative motion. Together, our studies point to some of the largest potential sources for measurement error when deploying instrumented mouthguards, namely lower jaw interference and decoupling.

Decoupling error is related to impact location, possibly because the headform is forced *into* the mouthguard in mostly rearward (rear and rear boss) impacts but *away* from the teeth in mostly frontal (front and front boss) impacts. This is especially noteworthy because a majority of impacts collected with mouthpieces in some studies are to the front of the head [31, 32]. The results of the present study agree with previous analyses that error depends on impact location and frontal impacts are generally characterized by greater error than rear impacts [12]. Interestingly, even when severe decoupling occurs, head kinematic data with errors similar to the no-gap condition can be collected in some impacts for a short period of the collection window. Average error decreases across all kinematic measures when the measurement window is limited to the impact duration in decoupling events. The windows we used (5.5 ms for rigid impacts and 12 ms for padded impacts) were not optimized, and may not represent the ideal signal window in decoupling events. Additionally, decoupling events collected in the field using similar devices may have different time windows during which the kinematics are valid.

Some level of significant proportional bias existed for most mouthpiece measurements in this study. Bias was of greater magnitude in open-mouth cases than in tight conditions, evidenced by WLP slope estimates drifting further from a value of one. Meaningful proportional bias was seen most often in open-mouth conditions. Fixed bias was of higher magnitude and more often significant in gap conditions. Padded impacts showed trends more clearly, likely because of the missing high-severity rigid impacts not in the dataset due to mouthguard recording errors, primarily frontal high-magnitude impacts (Table 1). Point estimates for WLP slope and intercept in nearly all tight conditions were closer to the target values (1 for slope or 0 for intercept) than corresponding point estimates for gap conditions. Most estimates moved closer to the target value when windowing the signals to the impact duration, suggesting that there is a window during which peak kinematics are valid, even for some severe decoupling events.

Decoupling signals were quantified as relative linear acceleration or angular velocity in the headform frame of reference at the teeth. Characteristic signals, differing by impact locations, were observed when relative motion occurred. Examining frequency-domain transformations of these signals also revealed a range of frequencies (approximately 100–500 Hz) excited excessively when relative motion occurred. Kuo et al. found that jaw motion created error frequencies with an average of 87 Hz in angular gyroscopes when compared to ATD measurements. His value is slightly lower than the range we identified as having excessive frequency content in acceleration measurements from decoupling, suggesting that jaw motion and decoupling may need to be dealt with via different data post-processing methods.

Our identified decoupling frequency range is greater than the greatest magnitude frequency observed via lab instrumentation in padded impacts from this study (approximately 50 Hz) but does overlap with peak frequency content from rigid impacts (approximately 150 Hz). In the future, these decoupling frequencies could be eliminated with more aggressive low-pass filtering, but an understanding of the effect of this filtering on reported peak kinematics, especially in high-frequency rigid impacts, must accompany this data-cleaning step to ensure peak kinematic reporting accuracy is not sacrificed in an attempt to salvage contaminated signals [27].

**Fig. 9:**
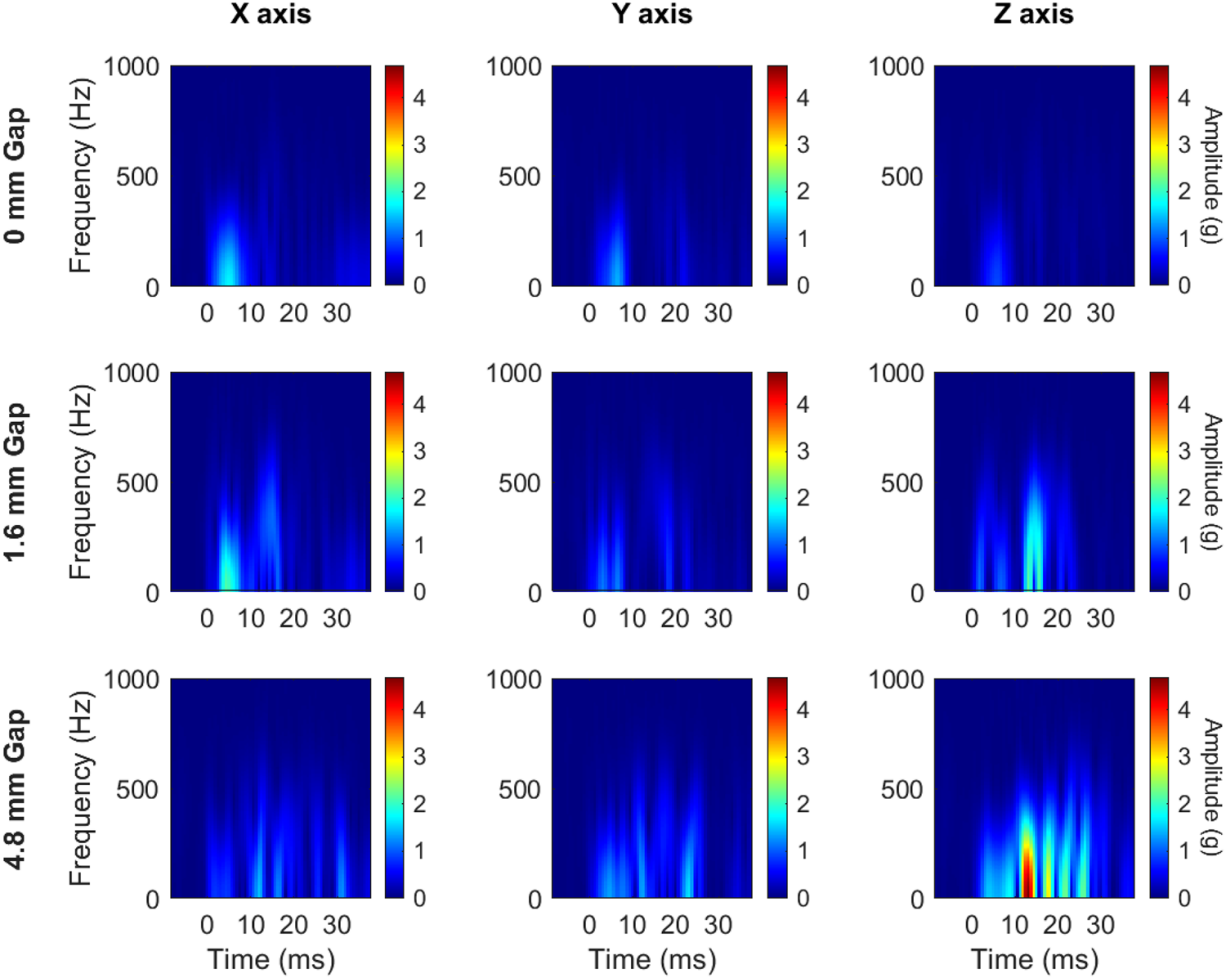
Short-time Fourier Transform of the relative linear acceleration signals (mouthguard minus CG transformed to teeth) along each axis from the 100 g padded front boss impact. The post-impact frequency content was progressively more noticeable with increasing gap size, especially along the *z*-axis for linear acceleration

**Fig. 12:**
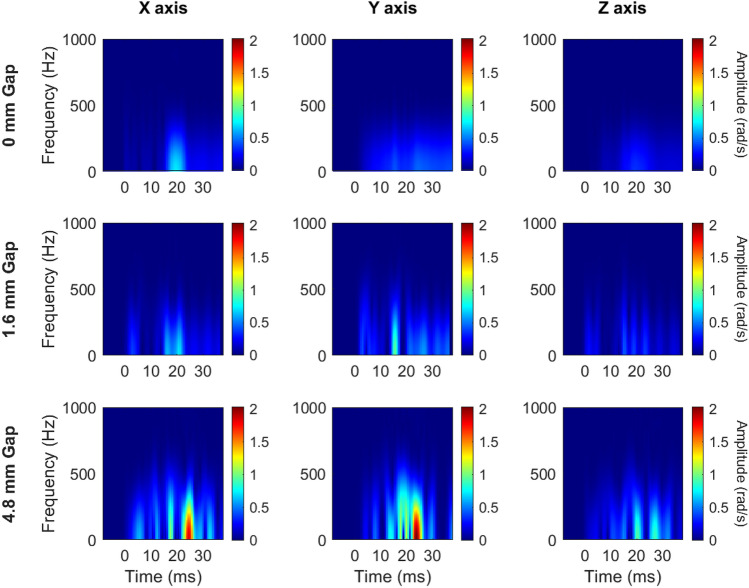
Short-time Fourier Transform of the relative angular velocity signals (mouthguard minus CG transformed to teeth) along each axis from the 100 g padded front boss impact. The post-impact frequency content in the *x*- and *y*-axes was progressively of higher amplitude with increasing gap size

**Fig. 13:**
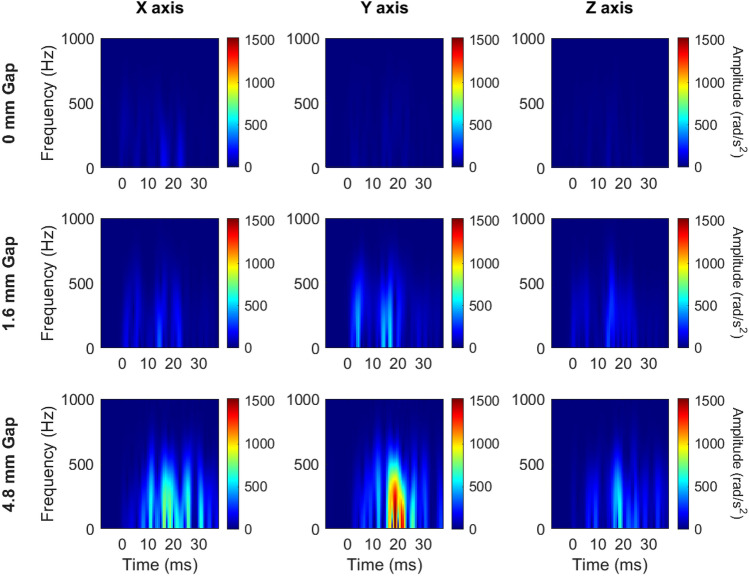
Short-time Fourier Transform of the relative angular acceleration signals (mouthguard minus CG transformed to teeth) along each axis from the 100 g padded front boss impact. Like angular velocity, the post-impact frequency content in the *x*- and *y*-axes was progressively of higher amplitude with increasing gap size

Despite elevated amplitudes in decoupling impacts, there was substantial overlap between decoupling and non-decoupling events in frequency content. As a supplementary analysis, we decided to generate short-time Fourier Transforms (STFT, MATLAB: *stft*) from the relative motion signals to explore if analyzing frequency with respect to time could better separate the decoupling signals from the tight condition. We applied a Hamming window to the zero-padded signals and allowed for 90% overlap in the time domain, then plotted signals up to the Nyquist frequency. As an exemplar impact, the most severe padded front boss impact (100 g target linear acceleration) is shown (Fig. 9). Time-frequency analysis revealed an increase in frequency magnitude toward the latter half of the data collection window in gap conditions after the linear acceleration had dropped to zero via the reference measurement at the headform CG. This frequency spike was most pronounced along the *Z* axis in linear acceleration signals and *X* and *Y* axes in angular signals (Figs. 12, 13, Appendix 2), which matched the pattern observed in the FFT plots. This pattern is physically consistent with what we would expect during a decoupling event: as the mouthguard is forced off the teeth it moves downward (*Z* axis, linear) relative to the teeth. In addition, some parts of the mouthguard would likely separate from the teeth before other parts (e.g., the mouthguard slips off the front of the teeth before the rear, or the right molars before the left), inducing a rotation about the *X* or *Y* axes. Thus, the frequency content of the relative motion signals describes decoupling distinctly, and the frequency content can be more clearly separated when considering frequency with respect to time.

The presented analyses may help to explain on-field artifacts that continue to plague instrumented mouthpiece studies [8]. Any studies using mouthpieces where relative motion is possible, suspected, or not described should be interpreted with the knowledge that measurement error may affect these data. Falsely high data may exist within the dataset, and efforts should be made to remove or salvage impacts with motion artifacts. In addition, falsely low data may exist within the dataset if over-filtering is employed without first understanding the effects of filtering on accuracy. Improvements to current systems are warranted, as even one of the best performing instrumented mouthguards on the market [8, 9] reported most impacts in this study as valid measurements despite the large amounts of relative motion observed.

Our study has several limitations. In our analyses, we included only impacts that were captured and reported by the mouthguard. Therefore, this study portrays the mouthguard’s capability when it performs as intended. Some rigid impacts were missed, resulting in data missing from our intended test matrix. These false negatives could have been included as zeros in our peak kinematics estimates, resulting in even greater errors in the mouthguard estimates for rigid impacts. However, because our rigid lab impacts represent edge cases that occur infrequently in the field, we chose to drop the missed impact conditions to show the capabilities of the mouthguard in the impacts it did capture. Thresholding effects have been shown to introduce bias to the triggering of instrumented mouthguards [33, 34] and the debate is ongoing to find the most optimal triggering solution for these devices. However, we do not necessarily believe that triggering bias was the primary cause of the mouthguard missing impacts in our study because the same acceleration peaks had different outcomes in rigid impacts than in padded. Location effects may play a role, as more front impacts were missed than rear.

Additionally, we used a lower dentition-to-mouthguard gap to evoke decoupling in this study. This successfully generated a range of mouthguard motion relative to the teeth, but decoupling events in the field could differ in their genesis and affects. For example, we did not specifically test how wear-and-tear may degrade mouthguard fit and create decoupling over time. Our tested conditions likely represent an extreme case for mouthguard decoupling. We saw several mouthguards leave the mouth entirely after the data collection window was over. While we confirmed with high-speed video that these occurrences did not affect our measurements, they do point to the severity of our testing and the marginal nature of our mouthguard’s fit. We used a single, commercially available instrumented mouthguard brand. While the sensors in this mouthguard represent other commonly available brands, results could differ slightly with different brands. Finally, the loss of several rigid impacts, especially higher severity and larger gap conditions, makes patterns less recognizable in characteristic time history curves and introduces bias by location and/or severity in the rigid dataset. Still, rigid impacts exhibited relative motion errors dependent upon severity and location.

## Conclusions

This study builds upon previous work to better understand mouthguard interactions that could occur in the field. We have shown an in-depth analysis of decoupling in a boil-and-bite style instrumented mouthguard and provided characteristic signatures and frequency content of relative motion for the potential identification of this phenomenon in the field. Open mouth conditions increased errors in mouthguard kinematic measurements, even in a properly fit boil-and-bite mouthguard. Impact duration and location effects were observed, pointing to conditional decoupling characteristics. All of these factors should be understood as potential limitations of field data from instrumented mouthguards and dealt with appropriately via advanced post-processing techniques when possible. Relative motion and the measurement errors that result should be avoided whenever possible by researchers by using the best obtainable coupling. When better coupling is not possible or when examining historical data, attempts should be made to further process or remove impacts in which relative motion is suspected. Both researchers and manufacturers can use these data to better deploy and interpret data from instrumented mouthguards.

## Figures and Tables

**Fig. 10:**
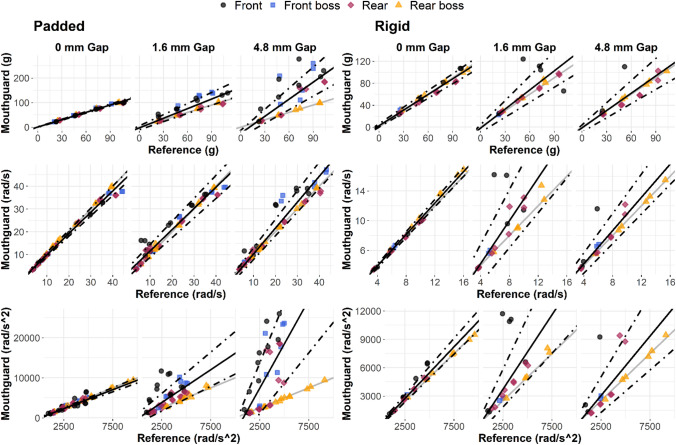
Mouthguard vs headform measurements for padded (left column) and rigid (right column) peak linear acceleration (top row), peak angular velocity (center row), and peak angular acceleration (bottom row), reported for the full measurement window. Solid lines are WLP estimates for slope. Dash-dotted lines are WLP 95% confidence intervals using a bootstrapping method. Gray solid lines represent a one-to-one relationship

**Fig. 11:**
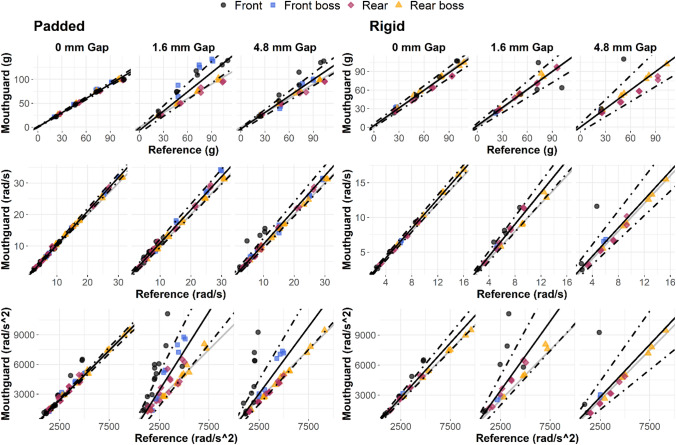
Mouthguard vs headform measurements for padded (left column) and rigid (right column) peak linear acceleration (top row), peak angular velocity (center row), and peak angular acceleration (bottom row), reported for the impact duration window. Impact durations: − 1 to 5 ms for rigid, − 1 to 12 ms for padded impacts. Solid lines are WLP estimates for slope. Dash-dotted lines are WLP 95% confidence intervals using a bootstrapping method. Gray solid lines represent a one-to-one relationship

**Fig. 14:**
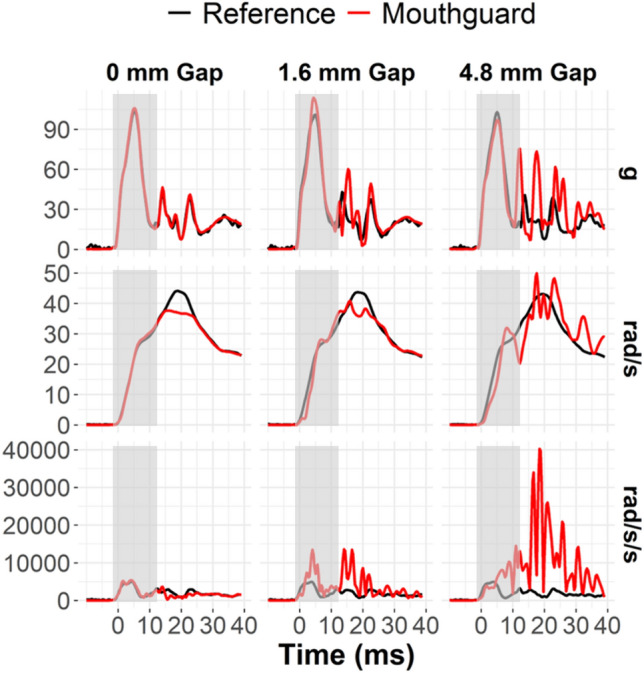
Exemplar time history plots of the kinematics from the 100 g padded front boss impacts. Reference curves were recorded at the CG and transformed to the teeth. Mouthguard curves were recorded at the teeth. We observed increasing oscillatory artifacts with greater gap conditions, but most of these artifacts occurred after the impact (gray boxes)

**Fig. 15:**
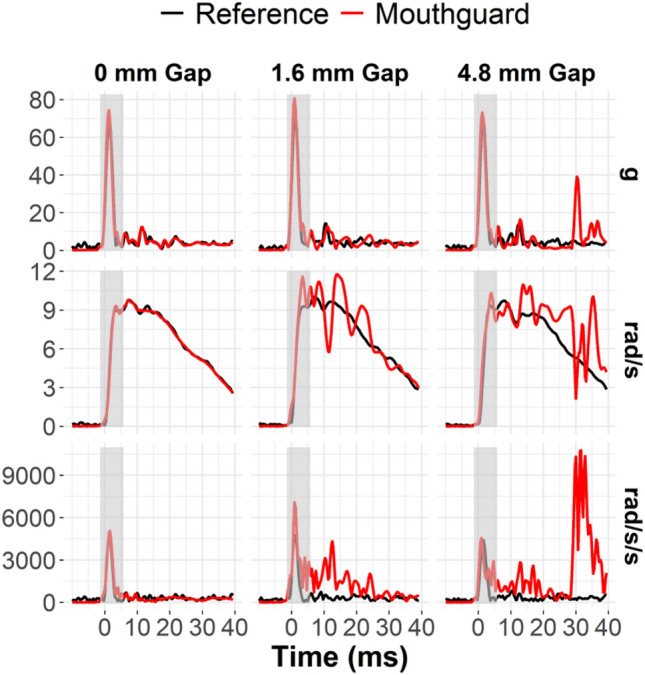
Exemplar time history plots of the kinematics from the 100 g rigid rear impacts. Reference curves were recorded at the CG and transformed to the teeth. Mouthguard curves were recorded at the teeth. We observed increasing oscillatory artifacts with greater gap conditions, but most of these artifacts occurred after the impact (gray boxes)

## Appendix 1: Weighted Least Products Scatter Plots

Weighted least products scatter plots are presented here, with lines and confidence intervals displayed with respect to a one-to-one line. Padded impacts show a clear trend of worsening bias with increasing gap size, while rigid impacts, which are sparser, show that gap conditions are generally worse than no gap (Fig. A1). When limiting the duration in which we searched for the peak (impact-windowed), we saw the fixed and proportional bias severity drop or disappear compared to the full duration analysis (Fig. A2). Differences are less obvious between gap conditions in the impact-windowed analysis, though angular acceleration still shows an obvious trend of worse bias with larger gaps.

Full Measurement Duration

See Fig. 10.

Impact-Windowed Duration

See Fig. 11.

## Appendix 2: Short Time Fourier Transform of Angular Acceleration

Frequency content with respect to time may be a very helpful analysis tool in analyzing mouthguard impacts where decoupling is suspected. Our study showed post-impact frequency spikes in rotation about the *X* and *Y* axes for relative angular signals made by the mouthguard (Fig. 12). These translated into similar spikes about the same axes for angular acceleration (Fig. 13). These areas of high frequency noise are most likely caused by the mouthguard separating from the front teeth more quickly than the molars (or vice versa) and from the impact side more quickly than the non-impact side, leading to rotation about the *X* and *Y* axes that worsens with increasing gap condition.

## Appendix 3: Example Time History Plots

Below, we provide example time history plots for select impact conditions (Figs. 14, 15). These plots demonstrate the relative accuracy of the mouthguard during the initial impact duration (gray boxes) and the artifacts present in the data thereafter for gap conditions. Impact duration was defined as − 1 ms to 12 ms for padded impacts and − 1 to 5.5 ms for rigid impacts.
